# Infrastructure in the Age of Pandemics: Utilizing Polypropylene-Based Mask Waste for Durable and Sustainable Road Pavements

**DOI:** 10.3390/polym15244624

**Published:** 2023-12-05

**Authors:** Nader Nciri, Namho Kim

**Affiliations:** 1 School of Industrial Design & Architectural Engineering, Korea University of Technology & Education, 1600 Chungjeol-ro, Byeongcheon-myeon, Dongnam-gu, Cheonan 31253, Chungnam, Republic of Korea; nader.nciri@koreatech.ac.kr; 2 School of Energy, Materials & Chemical Engineering, Korea University of Technology & Education, 1600 Chungjeol-ro, Byeongcheon-myeon, Dongnam-gu, Cheonan 31253, Chungnam, Republic of Korea

**Keywords:** sustainable infrastructure, pandemic mask waste recycling, asphalt modification, road pavement durability, sterilized shredded mask residues (SMRs), thermal resilience, rheological properties, dynamic shear rheometer (DSR), thermodynamic stability, rutting distress prevention

## Abstract

When navigating the environmental exigencies precipitated by global pandemics, the escalation of mask waste presents a multifaceted dilemma. In this avant-garde research, we unveil a novel approach: harnessing the sterilized shredded mask residues (SMRs), predominantly composed of 100 wt. % polypropylene, as pioneering modifiers for asphalt. Distinct proportions of SMR (e.g., 3, 6, and 9 wt. %) were judiciously integrated with fresh–virgin base AP-5 asphalt and subjected to an extensive suite of state-of-the-art examinations, encompassing thin-layer chromatography-flame ionization detection (TLC-FID), Fourier-transform infrared spectroscopy (FT-IR), scanning electron microscopy (SEM), thermogravimetric analysis (TGA), differential scanning calorimetry (DSC), and specific rheological metrics. The TLC-FID diagnostic trajectories highlighted the nuanced rejuvenating influence of SMR on the binder, a facet reinforced by a pronounced elevation in the thermodynamic stability index (I_C_). The FT-IR spectra elucidated SMR’s preeminent role as a filler, negating notions of chemical reactivity. The TGA analyses unveiled an elevated thermal onset of degradation, signposting enhanced thermal resilience, whereas the DSC readings illuminated a superior thermal comportment at lower extremities. The SEM evaluations rendered a clearer panorama: there was heightened textural perturbation at escalated SMR incorporations, yet the 3 wt. % concoction showcased an optimal, coherent microtexture symbiosis with asphalt. The rheological scrutinies revealed a systematic trajectory: a diminishing penetration and ductility countered by ascending softening points and viscosity metrics. The coup de maître stemmed from the DSR analyses, unequivocally validating SMR’s unparalleled prowess in curtailing rutting distress. This seminal inquiry not only posits a blueprint for refined pavement longevity but also champions a sustainable countermeasure to pandemic-propelled waste, epitomizing the confluence of environmental prudence an d infrastructural fortitude.

## 1. Introduction

In contemporary environmental and scientific discourse, the challenge posed by the escalating waste from face masks is at once unique and representative of broader systemic challenges [1,2]. Initially introduced as barriers against fine dust in places like South Korea and China [3,4,5,6], the prominence of face masks, due to the ongoing COVID-19 pandemic, has expanded to a global scale. This mass adoption, while crucial from a public health perspective, has inadvertently cast a spotlight on a burgeoning environmental quandary: the lifecycle and afterlife of billions of masks [7,8]. As we grapple with the ramifications of the pandemic, the specter of future virus waves or emergent pathogens serves as a clarion call, underscoring the urgency of preemptively addressing the environmental challenges intertwined with our health responses.

To understand the potential and challenges associated with mask waste, it is essential to delve deeper into the mask’s composition. A face mask, far from being a simple piece of cloth, is a marvel of modern material science [9,10]. Comprising multiple layers, with each serving a distinct purpose. Externally, masks are constructed for mechanical protection, often utilizing non-woven fabrics [11]. At their core, however, they incorporate specialized melt-blown polymers, acting as robust filtration systems [12]. These polymers, primarily polypropylene (PP), possess thermoplastic behaviors, characterized by their ability to be remolded under heat [13,14]. Alongside this, the chemical stability of polypropylene renders it durable in various environments [15]. The intricate interplay of these materials, from their fiber diameter and distribution to their porosity and permeability [16], raises intriguing possibilities for their recycling, particularly within specialized domains like road construction.

The field of road construction has long been at the forefront of pioneering recycling initiatives, driven by environmental imperatives. This endeavor is exemplified by the resourceful integration of diverse waste materials such as plastics [17], rubber [18], and even cooking oil [19] into road infrastructures. The transformative inclusion of polyethylene terephthalate (PET) in road pavements, for instance, has significantly enhanced their resistance to deformation, rutting, and water damage, marking a major advancement in pavement technology [20,21]. Similarly, the use of crumb rubber derived from recycled tires has proven to be a dual solution, addressing waste disposal issues while improving the elasticity and lifespan of asphalt roads [22].

Recent research has further broadened the scope of these endeavors. Zhao et al. have demonstrated the potential of disposable medical masks (DMMs) in improving asphalt performance [23]. By incorporating DMM waste, significant improvements in both the high- and low-temperature properties of asphalt were observed, pointing to a sustainable approach to managing pandemic-related waste [23]. Additionally, Xiao et al. have innovated a hydrophobic coating technology using waste plastic powder for coating acidic aggregates, thereby enhancing the moisture resistance of asphalt mixtures [24]. This technique is particularly significant in regions prone to moisture damage due to the use of problematic aggregates [24].

Building on these findings, the integration of high-density polyethylene (HDPE) into asphalt mixtures has been shown to improve their tensile strength and road durability, addressing the disposal problem of HDPE packaging waste [25]. Further, the addition of polyvinyl chloride (PVC), found in various consumer products, into asphalt mixes has been observed to increase a road’s resistance to heavy traffic wear and tear [26,27]. This not only solves the PVC waste issue but also reduces road maintenance requirements.

The use of polystyrene, a common packaging material, in road construction has also been explored. When mixed with bitumen, it acts as a lightweight aggregate, reducing road surface temperatures and mitigating urban heat islands [28,29]. Moreover, the incorporation of polypropylene enhances flexibility and crack resistance, which is beneficial in regions experiencing temperature extremes [30].

In addition to these, several other polymeric materials have shown promise. For example, the use of acrylonitrile butadiene styrene (ABS) plastic, known for its toughness and impact resistance, in asphalt mixtures can potentially enhance road durability in high-traffic areas [31,32]. Furthermore, research into utilizing polylactic acid (PLA), a biodegradable plastic, suggests that it could be used to increase the environmental sustainability of road infrastructures [33].

The success stories of these once-problematic wastes underscore the transformative potential of integrating mask waste into road infrastructures. Amidst this context, the layered architecture and distinct chemical attributes of masks stand out as potential candidates for enhancing asphalt mixtures. Polypropylene, with its notable tensile strength, hydrophobic properties, and chemical inertness [34], could provide asphalt with improved wear resistance, longevity, and reduced susceptibility to environmental degradation [35,36].

Yet, the narrative surrounding mask waste takes on a more urgent tone when viewed against the backdrop of the potential epidemiological challenges. With virologists and epidemiologists cautioning about the likelihood of future virus waves or novel pathogens [37,38], the prospect of an escalating reliance on masks becomes inescapable. Such scenarios not only emphasize the imperative to address the current waste challenges but also to anticipate and mitigate the environmental repercussions of future health crises.

Situated at the pivotal intersection of public health imperatives, environmental stewardship, and infrastructural innovation, this research undertakes a comprehensive and nuanced analysis of asphalt mixtures in conjunction with sterilized mask residues (SMRs). Our investigation rigorously examines the multifaceted dimensions of asphalt mixture properties—including but not limited to their chemistry, thermodynamics, micromorphology, physics, and rheology—and their intricate interactions with SMR. The overarching aim is to forge a groundbreaking, ecologically responsible, and resilient framework for infrastructure development, repurposing what is conventionally viewed as waste into a valuable, sustainable resource.

In this vein, our research represents an avant-garde foray into the realm of asphalt technology, innovatively incorporating SMR—a tangible byproduct of the pandemic crisis—into asphalt mixtures. This endeavor is characterized by a comprehensive examination of the interplay between SMR and asphalt across a spectrum of dimensions: chemical composition, thermodynamic stability, micromorphological configurations, physical robustness, and rheological characteristics. Notably, the incorporation of SMR into asphalt mixtures is facilitated through the application of a widely recognized and efficacious sterilization method: autoclaving. This choice underscores our commitment to utilizing established, reliable techniques to ensure the safe and sustainable integration of pandemic-related waste into infrastructure materials.

Our research findings are pivotal, revealing marked improvements in the structural durability, environmental sustainability, and overall functional quality of asphalt mixtures following the addition of a SMR. This breakthrough not only propels the field of sustainable infrastructure forward but also redefines the role of pandemic-related waste in material science and environmental engineering, positioning it as a valuable resource for cutting-edge solutions. Our interdisciplinary methodology, blending scientific precision with environmental foresight, represents a significant leap in the evolution of infrastructure suited to the challenges of a pandemic-affected world. Thus, this research marks a critical milestone in the journey towards innovative, durable, and sustainable infrastructure, embodying the synthesis of environmental responsibility and infrastructural ingenuity.

## 2. Materials and Methods

### 2.1. Transformative Integration: Harnessing KF94 Mask Residues for Advanced Asphalt Composite Formulation

In light of the profound implications of the global pandemic, contemporary scientific inquiries necessitate a recalibration, steering towards pragmatic solutions that bear global relevance yet are grounded in local contexts. This research elucidates such an approach, focusing on the methodical repurposing of KF94 masks, an item of paramount significance in South Korea’s pandemic response.

The KF94 mask, emblematic of South Korea’s engineering prowess in protective wear, derives its nomenclature from its intrinsic design metrics: “KF” is an abbreviation for “Korean Filter”, whilst “94” quantitatively underscores its capability to intercept 94% of airborne particulates as small as 0.3 microns, in strict adherence to South Korean regulatory benchmarks [39,40]. Notably, their ubiquity in the South Korean context, stemming from their widespread adoption as a primary protective measure, underscores their relevance and provides a substantive rationale for their selection in this investigative endeavor.

Given the potential biological ramifications associated with SARS-CoV-2, prudence mandated a deviation from sourcing these masks from conventional waste channels in Cheonan City, South Korea. Consequently, the study employed a repository of personally utilized KF94 masks that had been subjected to the rigors of high-density urban contexts.

The intricate constitution of the KF94 mask encompasses multiple layers, each meticulously designed for optimal filtration. For the ambit of this investigation, emphasis was placed solely on the three primary layers. Ancillary components, such as the metallic nose bridge and ear loops, although integral to the mask’s functionality, were judiciously excluded to ensure homogeneity in the shredded mask residues (SMRs) and to refine their synergistic compatibility with asphalt during subsequent amalgamation.

The detailed analysis of the KF94 mask components including their precise chemical compositions and their respective functions has been extensively laid out in Table 1. This table serves as an exhaustive repository delineating each layer’s role and the chemical substances integrated into their makeup, facilitating a comprehensive understanding of their individual and collective contributions to the overarching functionality of the mask.

Entrance into the investigative pipeline necessitated a rigorous quality assurance framework, engineered to rigorously screen out any specimens deviating from the established benchmarks, thus ensuring the utmost scientific integrity of the endeavor.

Post-validation, an exacting sterilization protocol was implemented, harnessing a high-pressure environment of 15 psi within an avant-garde steam autoclave (Hangil Biotech, Bucheon-si, Gyeonggi-do, Republic of Korea). This phase, characterized by a steadfast temperature regulation at 121 °C for a duration of 20 min, aligns with the apex of biomedical waste management standards [44], ensuring the comprehensive annihilation of potential biohazards and obviating the risks of cross-contamination.

Following sterilization, the masks underwent a methodical transformation phase, culminating in homogenized fragments with calibrated dimensions between 1 to 3 mm [45,46,47,48], facilitated by a bespoke mini-compact paper shredder (Fellowes Korea Co., Ltd., Seoul, Republic of Korea). This size range was selected based on prior assays for bitumen modification, which evidenced its potential for superior performance enhancements [45,46,47,48]. These SMRs, undeniably pivotal in the research trajectory, are primed for their forthcoming integration with asphalt substrates, aiming to reimagine and fortify their structural compositions.

**Figure 1:**
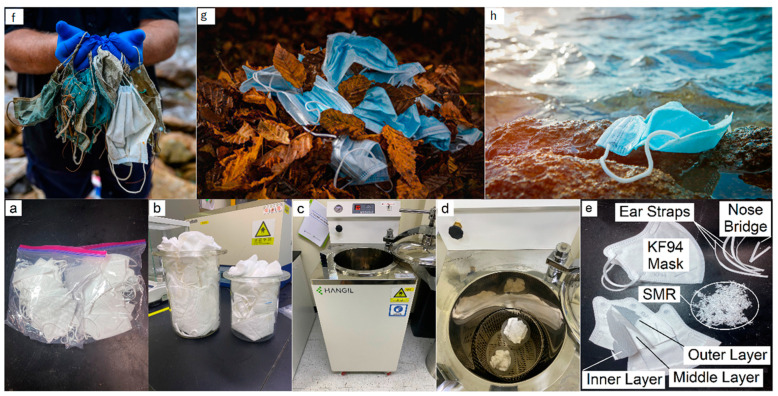
Methodology for deriving shredded mask residues (SMRs) from used KF94 masks. (**f**–**h**) Environmental context: Photographs highlight the issue of discarded coronavirus masks in open spaces, emphasizing environmental concerns. (**a**) Mask waste collection: The study utilized personally worn KF94 masks, with strict safety protocols due to potential contamination risks. (**b**–**d**) Sterilization process: Masks were autoclaved at 15 psi and 121 °C for 20 min, ensuring safety and decontamination. (**e**) Systematic disassembly of a KF94 mask and its conversion into shredded mask residues (SMRs, 1–3 mm) for potential asphalt performance enhancement.

For scholars and practitioners desiring an in-depth exploration of the nuanced methodologies employed, Figure 1 delineates the meticulous procedural architecture via a graphical schema, while Table 2 proffers a granular dissection of the chemical and physical attributes of the SMRs.

In synthesis, this research transcends being a mere academic exploration. It emerges as a confluence of environmental sagacity and engineering acumen, positing the transformative potential of KF94 masks, an emblem of South Korea’s pandemic response, in the augmentation of conventional asphalt substrates.

### 2.2. Formulation of Asphalt Composites with Varied Concentrations of Shredded Mask Residues: A Methodical Approach

Within the framework of this advanced exploration, a specific asphalt variant, denoted as AP-5 (PG 70–10), was utilized. This material was furnished by the eminent Ascon Industry Cooperative R&D Center based in Osan-si, Gyeonggi-do, Republic of Korea. Table 3 catalogs an analytical breakdown of its pivotal physical and chemical constituents. Concurrently, Figure 1e offers a nuanced portrayal of the shredded mask residues (SMRs), exactingly procured from a select ensemble of KF94 masks. This methodological approach emphasizes a calculated deviation from traditional waste sourcing, underscoring the importance of impeccable safety standards and experimental rigor. Table 2 provides a granular exposition of the SMR’s physicochemical facets.

To fabricate the AP-5–SMR composites, a GLHMD-B100 heating mantle (sourced from Global Lab Co., Ltd., Siheung-si, Republic of Korea) was calibrated to a temperature of 180 °C. An L5M-A high-shear mixer (Model No. GLHMD-B100, Silverson, East Longmeadow, MA, USA) operated at a speed of 3000 rpm was engaged for blending [51,52,53]. With an emphasis on curbing oxidation while sustaining fluidity, the binder experienced a gradual heating transition in the oven: starting from ambient conditions (approximately 25 °C) and reaching a temperature of 140 °C over a span of two hours. A cylindrical vessel was meticulously loaded with 600 g of this preheated asphalt, which was then further subjected to a heating protocol, elevating its temperature from 140 to 175 °C. Once the 175 °C mark was attained, shredded mask residues were integrated in measured proportions—specifically 3, 6, and 9 wt. % SMR relative to the cumulative mixture’s weight [51,52,53]. These particular concentrations were strategically chosen to facilitate an in-depth appraisal of the ensuing asphalt composite’s myriad of attributes—chemical, morphological, thermal, physical, and rheological. To ensure homogeneity in the mixture, a consistent mixing velocity of 3000 rpm was maintained for a duration of 2 h at 180 °C [51,52,53], aligning with the conventional temperature benchmarks for hot-mix asphalt (HMA) production. Post-mixing, these fabricated composites were securely stored within airtight metal containers, awaiting subsequent analysis.

### 2.3. A Comprehensive Analysis of Base AP-5 Asphalt Cement Composition Altered by Varying Concentrations of Discarded Shredded Masks Using TLC-FID Methodology

An exhaustive investigation employed thin-layer chromatography (TLC) in conjunction with flame ionization detection (FID) to examine the implications of using varying concentrations of discarded shredded masks (DSMs)—specifically 3, 6, and 9 wt. %—on the chemical composition of base AP-5 asphalt cement. The sophisticated apparatus incorporated a metallic assembly with Chromarod-S5 silica rods sourced from LSI Medience Corporation, Chiyoda-ku, Tokyo, Japan. Additionally, an IATROSCAN^®^ MK6s FID from Iatron Laboratories Incorporation, Tokyo, Japan, was utilized to ensure exact detection.

For the experiment, a 2 wt. % asphaltic solution was formulated by merging the binder (or SMR) with dichloromethane, which was subsequently applied to pre-activated and purified chromarods [54]. The subsequent phase involved segregating the binder into saturates, aromatics, and resins. This was achieved by immersing the chromarods sequentially in *n*-hexane for 45 min, toluene for 17 min, followed by a blend of dichloromethane and methanol in a 95:5 ratio for 5 min [54]. Post-separation, the SARA components (i.e., the saturates, aromatics, resins, and asphaltenes) underwent combustion, and the ensuing carbon ions were captured. SARA quantification was executed by integrating the four distinct zones on each silica gel bar. To ensure the removal of solvent residues, every chromarod holder was subjected to a drying phase at 85 °C for a duration of 2 min following each solvent development stage [54]. The analysis of each asphaltic sample was replicated four times to bolster the robustness and validity of the findings [54].

### 2.4. Assessing the Chemical Interplay of Base AP-5 Asphalt Cement and Varying Concentrations of Discarded Shredded Masks Using Fourier-Transform Infrared Spectroscopy (FT-IR)

In our study, we used Fourier-transform infrared spectroscopy (FT-IR) to probe the complex chemical dynamics arising from the amalgamation of the base AP-5 asphalt binder with varying concentrations of shredded mask residue (SMR)—specifically at 3, 6, and 9 wt. %. Each blend, reflecting these distinct concentrations, was subjected to a rigorous examination. The analysis involved 32 scans with a resolution of 1 cm^−1^, spanning a spectral range of 4000–650 cm^−1^, facilitated by the state-of-the-art Hyperion (3000 FT-IR) Spectrometer (Bruker Optics, Ettlinger, Germany). The acquired spectra were diligently normalized, with absorbance values cataloged to pinpoint the specific functional group interactions between the asphalt and the SMR. These detailed spectral data, when juxtaposed against the control asphalt blend, served to elucidate any chemical shifts or alterations induced by using varying concentrations of the mask materials.

### 2.5. The Microstructural Examination of Base AP-5 Bitumen Integrated with Shredded Mask Residues via Scanning Electron Microscopy (SEM)

In the pursuit of elucidating the intricate alterations in elemental composition and the nuanced shifts in the surface topography brought about by the incorporation of shredded mask residues at concentrations of 3, 6, and 9 wt. % SMR into the foundational AP-5 bitumen, a comprehensive analysis was conducted. This endeavor was facilitated by the prowess of the JSM-6010LA SEM (JEOL Ltd., Tokyo, Japan).

To enhance their innate electrical conductivities—a requisite for impeccable SEM imaging—the modified asphalt specimens were diligently immersed in liquid nitrogen (LN_2_, reaching a temperature trough of −80 °C). Subsequent to this cryogenic immersion, these specimens were delicately veneered with an ultra-fine gold film, approximately a thickness of 10 nm, leveraging the capabilities of an X sputter coater (Sputter Coater Model 108auto C3783, Cressington Scientific Instruments, England, UK).

In the acquisition of the SEM micrographs, a stringent imaging protocol was meticulously adhered to. For both the unmodified and SMR-modified asphalt specimens, a magnification of ×3000 was applied, whereas a magnification of ×50 was designated for the KF94 mask layers, encompassing the inner, middle, and outer strata. Concurrently, the process was characterized by a modulation with a beam current of precisely 5 nA, a consistently upheld working distance of 10 nm, and an operation executed under an accelerating voltage duly calibrated to 5 kV.

### 2.6. The Thermogravimetric Profiling of Base AP-5 Bitumen Augmented with Varied Concentrations of Shredded Mask Residues

Within the sophisticated realm of materials science, discerning the thermal dynamics of base AP-5 bitumen, particularly when it is nuanced by the integration of disparate concentrations of shredded mask residues (viz., 3, 6, and 9 wt. % SMR), emerges as a crucial endeavor. To dissect this intricate interplay, the state-of-the-art Thermogravimetric Analyzer (TGA Q500, TA Instruments, New Castle, DE, USA) was employed. Each thermogravimetric iteration necessitated a methodical elevation in temperature for a precisely aliquoted 10–15 mg of binder sample (infused with SMR), spanning a thermal gradient from 35 to 1000 °C. This controlled thermal progression was orchestrated at a consistent rate of 20 °C min^−1^, and executed within the purview of a nitrogen ambiance, ensuring an unwavering N_2_ flow rate of 150 mL min^−1^. To substantiate the robustness and reproducibility of the findings, this rigorous TGA protocol was reiterated on three separate occasions, underscoring an unwavering commitment to analytical precision and empirical integrity.

### 2.7. Calorimetric Investigations of Base AP-5 Bitumen Modified with Shredded Mask Residues via Differential Scanning Calorimetry (DSC)

Utilizing the sophisticated capabilities of the TA Instruments’ DSC apparatus (DSC Q20 V24.11 Build 124, New Castle, DE, USA), an extensive calorimetric analysis was executed to decipher the thermal nuances introduced to the pristine base AP-5 bitumen by incorporating varying fractions of shredded mask residues, specifically at concentrations of 3, 6, and 9 wt. % SMR. In a concerted effort to mitigate potential oxidative perturbations during the procedure, a meticulously quantified aliquot, approximating 5 mg of the modified asphalt sample, was enshrined within an aluminum crucible and subsequently ensconced in a nitrogen-enriched inert atmosphere. Employing a crucible devoid of any sample served as the control metric for the assay.

Initiating the protocol, the curated sample underwent a systematic thermal elevation from ambient temperature to +50 °C, progressing at a scanning cadence of 10 °C min^−1^. This phase spanned a temporal window of 10 min, ensuring a robust baseline measurement. Subsequent to this elevation, the sample experienced a thermal descent to a chilly −90 °C, again adhering to the 10 °C min^−1^ rate, only to be followed by an ascent to a +150 °C thermal zenith, paced similarly. These meticulous cooling and heating trajectories were devised to rigorously purge any residual thermal imprints from the bituminous specimens.

Post this inaugural thermal cycle, the sample was swiftly relegated from its +150 °C stature to the −90 °C nadir, where it lingered briefly for approximately 10 min. Thereafter, it embarked on its reheating journey at 10 °C min^−1^, culminating at +150 °C. This secondary heating scan facilitated the derivation of pivotal calorimetric indices, notably the enthalpy shifts (∆H) and the salient phase transition thresholds, such as the glass transition temperature (T_g_) and the melting temperature (T_m_).

To anchor the empirical rigor of this investigation, and thereby uphold the gold standard of reproducibility, the entire DSC assay protocol was reiterated thrice.

### 2.8. The Evaluation of Base AP-5 Asphalt Cement’s Properties under the Influence of Varied Shredded Mask Residue Concentrations: An Empirical Laboratory Examination

To comprehensively understand the impacts of distinct concentrations of shredded mask residue, specifically 3, 6, and 9 wt. % SMR, on the physical attributes of AP-5 asphalt cement in its unaged state, a systematic battery of conventional laboratory tests was devised and executed. These examinations, conducted in quadruplicate, encompassed assessments of the penetration, softening point, viscosity, and ductility.

#### 2.8.1. Penetration

In order to decipher the hardness characteristics of the various asphalt–SMR composites, a penetration test conforming to the ASTM D5 standard [55] is executed. The penetration apparatus or a Humboldt Mfg Electric Penetrometer (Humboldt Mfg. Co., Elgin, IL, USA) applies a load of 100 g through a standard needle onto the composite sample, maintained at a stable temperature of 25 °C, for a period of 5 s. The data obtained from this test forms the basis for determining how the fraction of mask material impacts the composite’s resistance to deformation, a critical attribute for assessing its suitability in bearing traffic loads. This process was repeated five times for each sample to ensure accuracy, and the average was reported.

#### 2.8.2. Softening Point

The ring and ball test apparatus RKA 5 (Anton Paar GmbH, Ashland, VA, USA), adhering to ASTM D36 standards [56], is employed to estimate the softening point of the asphalt–SMR composites. The method involves an evenly distributed heating of the sample until it reaches the softening point—defined as the temperature at which the asphalt can no longer bear the weight of a standard steel ball. This critical parameter provides insights into the composite’s temperature susceptibility and its ability to resist rutting at elevated temperatures. This process was repeated four times for each sample, and the average value was reported.

#### 2.8.3. Viscosity

The viscosity of the asphalt–SMR composites, a key parameter influencing the workability and compactibility of the asphalt mix, is evaluated using a Brookfield DV III Rotational Viscometer (Brookfield, Middleboro, MA, USA) according to ASTM D4402 standards [57]. The procedure involves heating the asphalt sample to a specified test temperature (typically 135 °C), after which the viscometer spindle (spindle number SC4-27) is rotated at a fixed speed of 20 rpm. The torque necessary to rotate the spindle is used to calculate the viscosity, providing vital information on the flow characteristics of the composite. This process was repeated four times for each sample, and the average viscosity was reported.

#### 2.8.4. Ductility

Lastly, the ductility of the asphalt–SMR composites is assessed using a ductilometer apparatus (Woojin Precision Co., Ltd., Gwangju-si, Republic of Korea), representing the asphalt’s ability to deform under tensile loads before breaking. As per ASTM D113 standards [58], a sample of the composite is pulled apart at a rate of 5 cm min^−1^ at a temperature of 25 °C until fracture occurs. This information is key to understanding the elasticity and flexibility of the asphalt–SMR composite and its resistance to cracking under load or temperature fluctuations. This process was repeated four times for each sample, and the average ductility was reported.

### 2.9. Thermorheological Characterization of Base AP-5 Asphalt via Penetrative Indices (PI and PVN) in the Presence of Shredded Mask Residues

To discerningly comprehend the thermorheological behavior of the base AP-5 bitumen upon the incorporation of discrete fractions of shredded mask residues (viz., 3, 6, and 9 wt. % SMR), the study engaged two sophisticated indices—the Penetration Index (PI) and the Penetration-Viscosity Number (PVN). Evidently, heightened values for both the PI and PVN are indicative of a binder’s fortified resilience against temperature-induced vicissitudes [59]. In a diametric scenario, diminished values are emblematic of a binder’s augmented susceptibility to temperature oscillations [59]. To be precise, binders with attenuated temperature sensitivity are poised to exhibit commendable viscosity metrics at elevated service temperatures, whilst maintaining requisite pliancy at subambient temperatures [59]. It warrants underscoring that, notwithstanding the astuteness of these determinative methodologies, they predominantly serve as canonical approximations for binder comportment. For an exhaustive, real-world extrapolation within the ambit of hot-mix pavements, data gleaned from the Dynamic Shear Rheometer (DSR), especially with its unwavering emphasis on the rutting factor in an unaged paradigm, is quintessential.

The Penetration Index (PI) is extrapolated as per the mathematical construct encapsulated in Equation (1):

(1) $$PI=\frac{1952-500\log P-20\times SP}{50\log P-SP-120}$$

wherein:

P: epitomizes the penetration, quantified in decimillimeters (dmm) and assayed under stipulated conditions [25 °C, 100 g, 5 s].

SP: is emblematic of the softening point, ascertained in Celsius (°C).

The Penetration-Viscosity Number (PVN) is derived pursuant to the relation in Equation (2):

(2) $$PVN=-1.5\frac{4.2580-0.7967\log P-\log V}{0.7591-0.1858\log P}$$

In this equation:

P: signifies the penetration magnitude, enumerated in decimillimeters (dmm), under the conditions [25 °C, 100 g, 5 s].

V: is representative of the kinematic viscosity, adjudged at 135 °C, and articulated in centistokes (cSt).

### 2.10. Rheological Evaluation of the Base AP-5 Asphalt Incorporating Shredded Mask Residues: Dynamic Shear Rheometer (DSR) Analysis

Utilizing a Dynamic Shear Rheometer (DSR) by ThermoFisher (Thermo Scientific^TM^ HAAKE^TM^ MARS^TM^ Rheometer, Thermo Fisher-Scientific, Newington, NH, USA), a comprehensive assessment was undertaken to elucidate the influence of varying proportions of shredded mask residues, notably 3, 6, and 9 wt. % SMR, on the viscoelastic behavior of the foundational AP-5 asphalt cement. This analysis exclusively concentrated on the rutting factor under unaged conditions, a paramount determinant in ascertaining the pavement’s susceptibility to deformations under traffic loading.

The DSR protocol was meticulously executed in alignment with ASTM D7175 [60]. Opting for a loading frequency of 10 rad s^−1^ (1.59 Hz) facilitated a simulation of shearing dynamics, analogous to a vehicular speed of approximately 55 mph (90 km h^−1^). Through the acquisition of the complex shear modulus |G*|—signifying stiffness, and the phase angle (δ)—indicative of potential plastic deformation, critical insights into the rutting factor (G*/sin δ) were obtained.

For the higher temperature bracket of 46–82 °C, which was the focal point for analyzing the rutting potential, an asphalt specimen exhibiting a thickness of 1 mm and a diameter spanning 25 mm was deemed appropriate.

## 3. Results and Discussion

### 3.1. Chemical Reconfiguration of Base AP-5 Asphalt with Shredded Mask Residues: Insights from TLC-FID Analysis and Colloidal Chemistry

In recent years, the modification of asphalt binders using diverse waste materials has surged to the forefront of pavement materials research. The quest for sustainable materials and a deeper understanding of binder chemistry has rekindled interest in the colloidal disposition of asphalt, and more specifically, its SARA (i.e., saturates, aromatics, resins, and asphaltenes) constituents.

This study deciphers the complex interplay between the introduction of shredded mask residues (SMRs) and the resultant shifts in the SARA composition of fresh–plain base AP-5 asphalt using the avant-garde thin-layer chromatography-flame ionization detection (TLC-FID) method.

**Figure 2:**
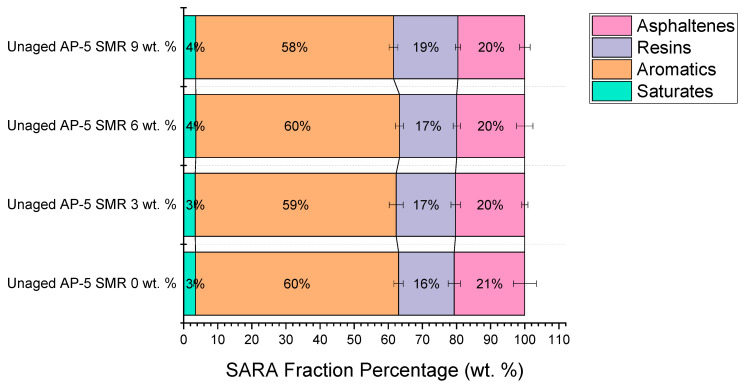
Modulation of SARA constituents in unaged base AP-5 bitumen with progressive integration of shredded mask residues (e.g., 3, 6, 9 wt. % SMR).

From the chromatograms procured via TLC-FID, showcased in Figure 2, the delineation of each constituent within the SARA framework was crystal clear. A paramount observation was the invariability of the saturates, which stood at approximately 3.47 ± 0.46 wt. % for the unmodified AP-5 binder and remained largely undisturbed even with incremental SMR, highlighting a range of 3.47 ± 0.46 to 3.57 ± 0.52 wt. %. This immutability underscores the low-polarity, non-reactive nature of the saturates, emblematic of their chemically inert dispositions within the colloidal asphaltene-resin matrix.

Conversely, the aromatic hydrocarbons exhibited a nuanced modulation. Starting at 59.52 ± 1.36 wt. % for the pristine AP-5, there was a discernible decrease to 57.97 ± 1.28 wt. % with a 9 wt. % SMR. Delving into the asphalt colloidal chemistry, it can be postulated that the aromatics, which play a pivotal role in maintaining the dispersion stability of asphaltenes, might be undergoing certain molecular rearrangements or solubilization due to their interactions with SMR constituents.

The most intriguing revelation was the enhancement in the resin’s fraction. The pristine AP-5 asphalt manifested a 16.30 ± 1.76 wt. %, which intriguingly escalated to an 18.87 ± 0.75 wt. % upon the incorporation of a 9 wt. % SMR. Given the SMR’s inherent composition being 100 wt. % resins, this augmentation is anticipated. In the intricate dance of colloidal chemistry, resins function as peptizing agents [61,62], ensuring that the high-molecular-weight asphaltenes remain stably dispersed [61,62]. Their amplified presence postulates an enriched polar matrix, potentially leading to an improved adhesion and resistance to moisture-induced damage.

Asphaltenes, the complex high-molecular-weight juggernauts of asphalt chemistry, indicated a decrement—from an initial 20.67 ± 3.35 wt. % in the unadulterated AP-5, they tapered off to 19.60 ± 1.56 wt. % with a 9 wt. % SMR. Colloidally speaking, the introduction of external resins could be inducing a reconfiguration of the native asphaltene-resin equilibrium, thereby suppressing the relative concentration of asphaltenes.

In synthesis, this analytical foray illuminates the intricate chemical transformations induced by SMR addition, offering profound insights into the colloidal and molecular realms of modified asphalt. Such revelations not only amplify our understanding of asphalt chemistry but also beckon innovative avenues for sustainable pavement engineering.

### 3.2. Enhanced Colloidal Stability of AP-5 Bituminous Binders with Shredded Mask Residues

The intricate balance of components in bituminous binders governs their colloidal stability, a key determinant of road pavement longevity. Understanding the Colloidal Instability Index (I_C_) or the Gaestel Index is imperative as it delves deep into the microscale stability of binders, portraying the equilibrium between stabilizing and destabilizing components [63,64,65].

Traditionally, bitumen is conceptualized as a colloidal system. Within this system, the aromatics coupled with resins operate as the backbone that stabilizes the asphaltenes. Conversely, the presence of saturates and certain asphaltenes can challenge this stability [66,67,68]. Drawing upon this understanding, the I_C_ is an eloquent representation of this balance, encapsulating the ratio of potential destabilizers (including flocculants like saturated oils and asphaltenes) to stabilizers (such as peptizers (resins) and solvents like aromatic oils) [66,67,68]. When delving into the specifics, an I_C_ value that is lower signifies a more stable presence of asphaltenes in the binder, with values less than or equal to 0.7 indicating a robust stabilizing influence predominantly from the resins [66,67,68].

**Figure 3:**
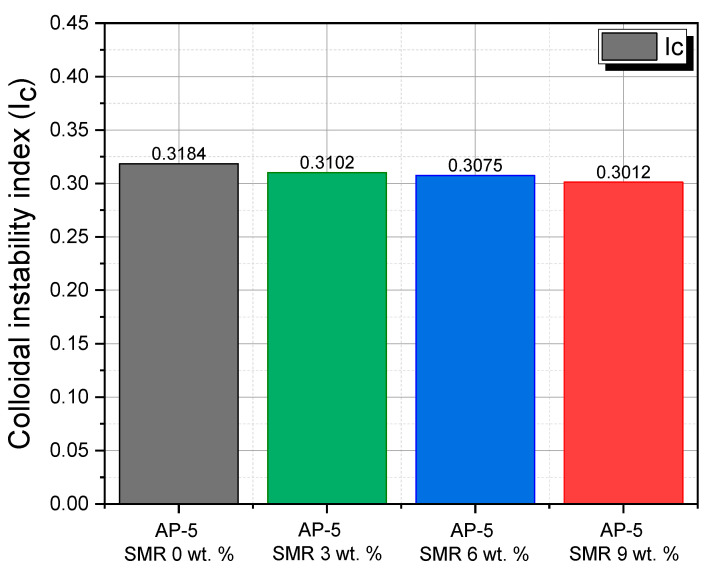
Impact of incremental concentrations of shredded mask residues (e.g., 3, 6, and 9 wt. % SMR) on the colloidal stability index (I_C_) of pristine base AP-5 asphalt.

Analyzing the provided data, as depicted in Figure 3, brings some insights into sharp relief: The inherent stability of AP-5 with a 0 wt. % SMR is evident from its lowest I_C_ value of 0.3184. The introduction of the SMR, which is purely resinous, into the binder intensifies this stability. With an addition of a 3 wt. % SMR, the I_C_ subtly decreases to 0.3102. As we progressively increase the SMR content to 6 wt. % and 9 wt. %, we observe a consistent and gentle reduction in I_C_ to 0.3075 and 0.3012, respectively. This trend reaffirms the quintessential role of resins in asphalt chemistry. Resins, functioning as peptizing agents, promote a more even dispersion of asphaltenes, which culminates in a heightened stability of the binder [69].

Diving deeper into the colloidal intricacies of bitumen, we can imagine it as a meticulously structured suspension. Within this suspension, a plethora of micelles, each with its own resinous shell and an asphaltene core, is uniformly dispersed in a continuous phase, primarily composed of aromatics and saturates. It is the synergistic interplay among these components that outlines the binder’s overarching rheological properties [70,71,72,73].

A detailed examination presents three distinct classifications:

(1) Sol-bitumina (I_C_ ≤ 0.7): Characterized by a predominant resin and oil content and subdued asphaltene presence, these binders display a pronounced liquid consistency. Their defining characteristics are a markedly reduced viscosity coupled with a heightened plasticity and temperature susceptibility. The capacity for self-healing post-damage further accentuates their appeal [70,71,72,73]. The samples furnished, regardless of the SMR content, squarely fit this description. The unerring decline in I_C_, even at elevated SMR concentrations, underscores the contribution of SMR toward enhancing asphaltene stability.
(2) Sol-Gel bitumina (0.7 < I_C_ < 1.2): These exhibit a balanced proportion of asphaltenes, which engenders a dual behavior, both elastic and viscous. The escalation in micelle concentration directly translates to an amplified plastic deformation. Their exemplary rheological characteristics earmark them as prime candidates for road construction [70,71,72,73].
(3) Gel-bitumina (1.2 < I_C_): Dominated by a systematic array of asphaltene micelles, these binders are renowned for their high viscosity. Their diminished plasticity, paired with their gel-like structure, earmarks them for specialized architectural endeavors [70,71,72,73].

In summation, infusing SMR into AP-5 not only fortifies the colloidal stability of the binder but also underscores the transformative potential of resins as stabilizers in bituminous binders. This revelation could potentially revolutionize road construction, offering an innovative avenue to recycle mask residues. However, the holistic evaluation of this approach would necessitate a deeper dive, examining its long-term performance and ecological implications.

### 3.3. Fourier-Transform Infrared Spectroscopy (FT-IR) Insights into Base AP-5 Asphalt Modified with Shredded Mask Residues

The Fourier-transform infrared spectroscopy (FT-IR) analysis offers profound insights into the potential repercussions of assimilating distinct concentrations of shredded mask residues (SMRs)—ranging from 3 to 9 wt. %—into the multifaceted molecular composition of pristine base AP-5 asphalt.

**Figure 4:**
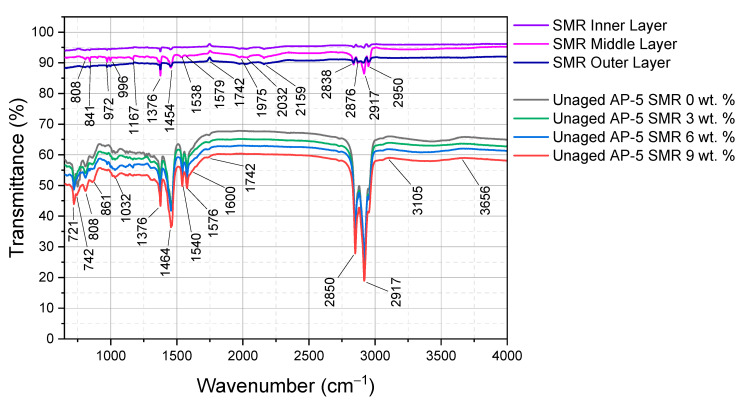
Comparative FT-IR spectra illustrating the distinct layers of the KF94 mask (i.e., outer, middle, and inner) alongside the fresh foundational AP-5 bitumen and its blends enriched with various concentrations of shredded mask residues (e.g., 3, 6, 9 wt. % SMR).

In Figure 4, the FT-IR spectrum of fresh–plain AP-5 asphalt (i.e., Unaged AP-5 SMR 0 wt. %) paints a vivid tableau of molecular interplay [74,75,76]. Within the 2800–3000 cm^−1^ region, aliphatic hydrocarbons are pronounced, marked by stretches typical of the C–H bonds. This range primarily mirrors the organic framework of asphalt, bestowing it with both pliability and robustness. Adjacently, a prominent band in the 3105–3656 cm^−1^ range, typically associated with O–H and N–H stretches, suggests the presence of potential hydroxyl or amine groups. These groups might be responsible for the asphalt’s capacity to interact with external agents or undergo oxidation processes, affecting its long-term performance. At 1742 cm^−1^, a tiny shoulder possibly corresponding to carbonyl functionalities emerges, highlighting the inclusion of esters or ketones in the asphalt. Peaks at 1576 cm^−1^ and 1540 cm^−1^ allude to the aromatic hydrocarbons, replete with delocalized electrons, fortifying the asphalt’s molecular structure and providing cohesion and strength. The spectrum’s nuances expand with indicators at 1450–1500 cm^−1^ and 1030–1100 cm^−1^, denoting alkyl groups and sulfoxides, respectively. Additionally, characteristic peaks at 742 cm^−1^, 808 cm^−1^, and 861 cm^−1^ underline the presence of specific substituted aromatic structures, broadening our grasp of the asphalt’s chemical fabric.

Upon analyzing the shredded mask residues (i.e., SMRs) through the lens of the FT-IR spectrum portrayed in Figure 4, the chemical composition of each layer, predominantly characterized by polypropylene elements, becomes discernibly distinct [77,78,79]. The outer layer is imprinted with stretches around 2950 cm^−1^, 2917 cm^−1^, and 2876 cm^−1^, likely corresponding to –CH_3_, –CH_2_, and –CH stretches, respectively. These suggest the presence of diverse hydrocarbons, reinforcing its water-resistant attributes. Peaks at 2159 cm^−1^ and 2032 cm^−1^, typically earmarked for nitrile or isocyanate functionalities, and carbonyl stretches at 1742 cm^−1^, intimate an elaborate polymer matrix, potentially contributing to the layer’s mechanical integrity. The middle layer unfolds with N–H stretches around 1538 cm^−1^ and 1579 cm^−1^, indicative of polyamide structures or analogous nitrogen-abundant polymers. Its filtering capabilities are possibly accentuated by peaks around 1975 cm^−1^. The innermost layer, tailored for comfort, radiates with stretches at 1167 cm^−1^, 996 cm^−1^, and 972 cm^−1^, resonating with C–O–C, suggestive of ether bonds, possibly providing a soft and pliable feel. Peaks at 841 cm^−1^ and 808 cm^−1^ further demystify the complex polymeric nature of this layer.

Synthesizing these findings, a salient observation is the unperturbed spectral integrity of the base AP-5 asphalt, even amidst graded introductions of SMR. The AP-5’s chemical milieu exhibits consistency, advocating a primarily physical incorporation of the SMR over the chemical amalgamations. This underlines the role of SMR as a non-reactive filler rather than a dynamic modifier. Extrapolating these insights, we can theorize that asphalt composites augmented with mask residues may leverage the intrinsic chemical sturdiness of the masks. Consequently, this can furnish the binder with superior mechanical traits while safeguarding its inherent chemical robustness—a significant boon for asphalt applications in ever-evolving urban settings.

### 3.4. Microstructural Dynamics of Base AP-5 Asphalt with Progressive SMR Additions: An Interdisciplinary Evaluation Using SEM, FT-IR, and SARA Techniques

Embarking on this study, our objective was unequivocal: to discern the intricate morphological, topographical, and microstructural changes in the fresh–virgin base AP-5 asphalt upon the systematic inclusion of shredded mask residues (e.g., 3, 6, 9 wt. % SMR), extracted from the tri-layered KF94 mask.

**Figure 5:**
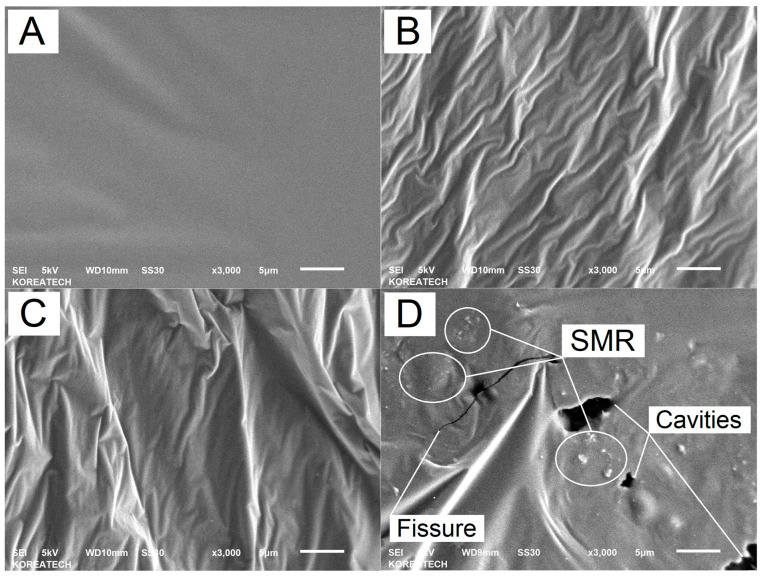
Scanning electron microscopy (SEM) photomicrographs showcasing pristine base AP-5 asphalt juxtaposed with variations incorporating discrete SMR percentages taken at ×3000 magnification. (**A**) Fresh–virgin base AP-5 asphalt; (**B**) Base AP-5 asphalt supplemented with a 3 wt. % SMR; (**C**) Base AP-5 asphalt integrated with a 6 wt. % SMR; (**D**) Base AP-5 asphalt enriched with a 9 wt. % SMR.

The foundational state of the base AP-5 asphalt, impeccably captured in Figure 5A, reveals a microsurface that is a paradigm of uniformity. Its unblemished, even topography, devoid of any microstructural aberrations, offers a pristine canvas against which the progressive alterations introduced by SMRs can be judiciously assessed.

**Figure 6:**
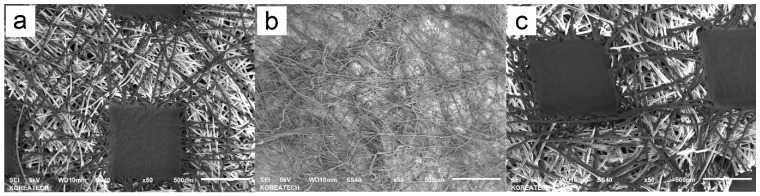
Scanning electron microscopy (SEM) micrographs of the KF94 mask layers at ×50 magnification. (**a**) Outer layer characterized by its robust structure for moisture resistance and durability. (**b**) Middle layer, the primary filter medium with a dense intertwined fiber matrix for optimal filtration. (**c**) Inner layer displaying a fibrous network for comfort and breathability.

Delving deeper into the intrinsic attributes of the KF94 mask, one uncovers a myriad of structural nuances embedded within its tri-layer composition. The outermost layer, distinctly visualized in Figure 6a, predominantly comprises spunbond polypropylene (PP). This layer, with its intricate polymeric weave, is engineered not merely for structural tenacity but also to offer tactile finesse. Conversely, the inner comfort layer, delineated in Figure 6c, exudes a subtler microtexture, emphasizing the mask’s commitment to user-centric comfort. The core layer, or the middle filtration-centric layer, captured in Figure 6b, is a dense tableau of melt-blown polymers. Each polymer strand here is purposefully positioned, underscoring its primary directive: the meticulous filtration of airborne particulates.

Upon meticulous examination through scanning electron microscopy (SEM) at a 3000-fold magnification, the microstructural evolution of AP-5 asphalt with incremental inclusions of shredded mask residues (SMRs) is vividly delineated. Figure 5 illustrates a series of SEM photomicrographs that capture the incremental textural transformations within the fresh–virgin AP-5 asphalt matrix upon the progressive addition of the SMR at concentrations of 0, 3, 6, and 9 weight percent.

The baseline condition of the fresh–virgin AP-5 asphalt, as depicted in Figure 5A, presents a homogenous and unblemished microsurface, indicative of an unaltered state. The inclusion of a 3 wt. % SMR, as observed in Figure 5B, introduces subtle topographical variations. These are characterized by mild, wave-like undulations across the asphalt’s surface, evocative of a tranquilly rippled aqueous surface. Despite this, the asphalt retains a largely uniform texture with no discernible SMR particulates.

Elevating the SMR content to a 6 wt. % engenders a more pronounced morphological transition, as evidenced in Figure 5C. The asphalt’s microsurface exhibits increased tortuosity, with the emergence of a crinkled texture that signifies a departure from the initial smoothness. The waveform patterns are now more distinct, yet the direct imprint of the SMR remains elusive within the microstructural tableau.

The culmination of SMR incorporation at a 9 wt. %, as portrayed in Figure 5D, marks a significant departure from the preceding states. The microstructure is characterized by a labyrinth of microstructural distortions, intermingled with discernible fissures and cavities. The SMR fragments now assert their presence unequivocally, punctuating the surface and reflecting a material system that is approaching the threshold of its microstructural accommodation capacity.

In summation, this SEM analysis substantiates that the incremental inclusion of SMR profoundly influences the microstructural landscape of AP-5 asphalt. The progression from a smooth to a complex topography with observable fissures and voids suggests a threshold beyond which the addition of SMR may compromise the integrity of the asphalt’s microstructure. The SEM images serve as a testament to the delicate balance between material modification and structural performance.

Reinforcing our SEM observations, the Fourier-transform infrared spectroscopy (FT-IR) analysis affirms that the SMR predominantly functions as a physical filler, with little to no chemical interplay with the asphalt. This lack of a chemical liaison was further corroborated by the absence of indicative spectral peaks suggesting any polymer-asphalt interactions.

Augmenting this analytical narrative, the thin-layer chromatography flame-ionization detection (TLC-FID) offers a granular insight into the molecular shifts. With incremental SMR additions, the saturates, which are relatively chemically inert, exhibit minimal fluctuations. In stark contrast, aromatics exhibit a diminishing trajectory, whereas resins manifest a pronounced surge. Concurrently, asphaltenes follow a regressive arc. Such molecular reconfigurations, potentially driven by the presence of SMR, signify their profound role in modulating the composite’s molecular equilibrium.

In real-world terms, the implications are profound. As urban landscapes evolve, the potential repurposing of waste materials, like SMRs, in infrastructural frameworks such as asphalts, can significantly dictate the performance and longevity of our roadways. Being cognizant of these microstructural dynamics is imperative for informed engineering decisions in the future.

In conclusion, our analytical odyssey, leveraging SEM, FT-IR, and TLC-FID, has meticulously chronicled the transformative journey of AP-5 asphalt with SMR integrations. This exploration not only amplifies the complex interplay between microscopic and macroscopic domains but also outlines the critical thresholds of material harmonization, invaluable for cutting-edge asphalt engineering endeavors.

### 3.5. The Thermogravimetric Analysis (TGA) of Shredded Mask Residues Incorporated in Pristine Base AP-5 Asphalt

The Thermogravimetric Analysis (TGA), coupled with its derivative method (DTGA), provides profound insights into the intricate thermal behaviors of materials. In the context of asphalts, especially when incorporated with novel additives such as shredded mask residues (SMRs), this analytical technique serves as a compass, guiding us through the myriad of thermal events these blends undergo.

**Figure 7:**
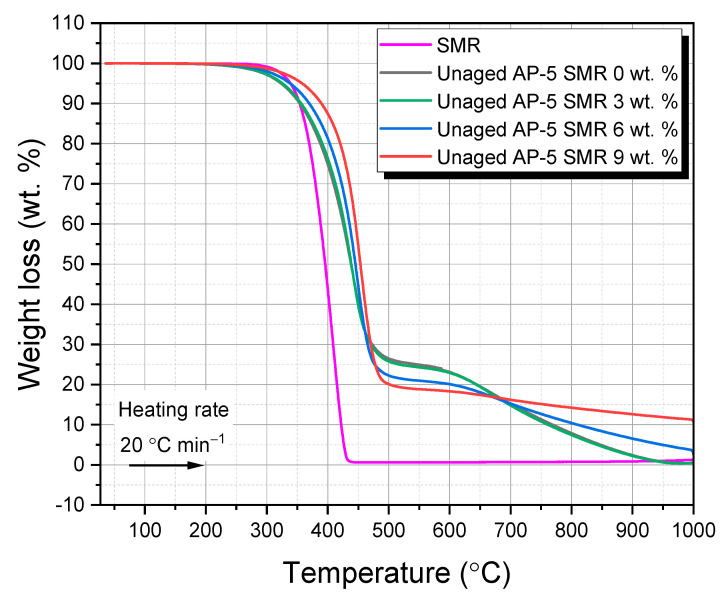
TGA profiles of shredded mask residues (SMRs), unmodified base AP-5 asphalt, and blends encompassing varied concentrations of SMR (e.g., 3, 6, and 9 wt. %).

**Figure 8:**
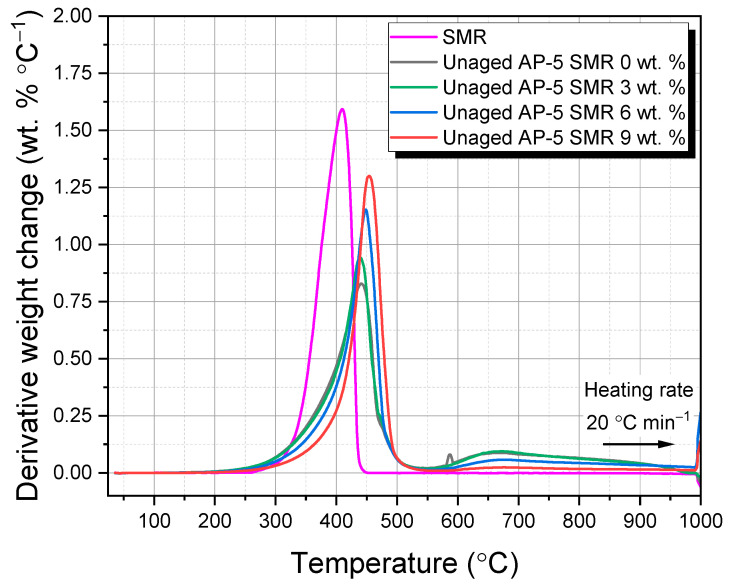
DTGA profiles of shredded mask residues (SMRs), pristine base AP-5 asphalt, and formulations containing diverse loadings of SMR (e.g., 3, 6, and 9 wt. %).

Figure 7 and Figure 8 meticulously chart the primary and derivative thermograms, plotting the weight percentage against the temperature. They offer a comparative panorama of the SMR, virgin bitumen, and a spectrum of unaged AP-5 asphalt blends integrated with varying SMR proportions (e.g., 3, 6, and 9 wt. %).

Figure 7 unfurls the thermal narrative of the fresh–virgin base AP-5 asphalt (i.e., Unaged AP-5 SMR 0 wt. %), subjected to incremental temperature hikes [80,81,82]. The onset of its primary thermal perturbation, demarcated at 378.55 °C, offers a barometer of its thermal resilience. This thermal journey can be compartmentalized into three distinct regimes: (1) the evaporation of light hydrocarbons (36.15–378.55 °C), (2) a more aggressive degradation of complex hydrocarbon chains (378.55–463.62 °C), and (3) the culmination of the robust asphaltene constituents capitulating to high temperatures (463.62–999.95 °C). Figure 8, on the other hand, unveils an intriguing, singular decomposition pattern, peaking at 433.88 °C. This suggests a dominant aromatic character of the native bitumen, a hypothesis further corroborated by ancillary TLC-FID investigations.

Delving into the SMR’s TGA profile, as presented in Figure 7, one discerns three salient stages of weight loss, reflective of its intricate multi-layered and multi-component construct [78,79,83,84]. The initial phase (*i*), spanning 36.02–364.95 °C, predominantly pertains to the evaporation of superficial moisture and the loss of volatile components embedded within the mask layers. These could be attributed to the superficial treatments and minor additives imparted to the mask for comfort and finish.

Transitioning to the intermediate stage (*ii*) (364.95–427.36 °C), a stark 99.27 wt. % mass depletion is observed. This loss can be construed as the thermal degradation of the primary synthetic polymers, likely the polypropylene or similar constituents, which form the structural backbone of the mask. This observation is further buttressed by one pronounced thermal event in DTGA’s SMR at 409.72 °C, a hallmark of the diverse synthetic polymers’ decomposition sequences.

The terminal thermolytic realm (*iii*) (427.36–575.49 °C) delves into the combustion of the residual polymers and tertiary additives, which might include certain elastic components or microbial treatments. Beyond the 575.49 °C threshold, the majority of the constituents capitulate to pyrolysis, bequeathing an ash residue, which, as our data suggests, lingers at around 1.49 wt. % as we approach 999.96 °C.

The inherent thermal fortitude manifested by SMR presents a compelling narrative for its prospective adoption in road engineering. Particularly, when one contemplates the thermal rigors the SMR–bitumen amalgams would be subjected to during Hot-Mix Asphalt (HMA) procedures. This resilience not only stands as a testament to the mask’s intrinsic polymer matrix but also hints at its potential symbiosis with bitumen.

Upon a thorough analysis of the data presented in Table 4, the incorporation of SMR into the AP-5 asphalt matrix underscores a noteworthy thermal transformation. This evolution, characterized by the marked increase in the onset degradation temperature (T_onset_), holds significant implications for real-world applications.

An elevated onset degradation temperature suggests that the asphalt mix, fortified with SMR, may sustain higher temperatures before initiating degradation. In real-world asphalt production and paving operations, this heightened thermal resilience has the potential to expand the window of workability, allowing for extended laydown times, especially in warmer climates or during peak summer months.

Moreover, with an extended temperature range before degradation begins, the risk of premature aging or hardening of the binder diminishes. This prolongation could lead to a more durable and long-lasting pavement, reducing the frequency and cost of its maintenance or replacements.

Furthermore, in the context of energy efficiency, a mix that is resilient to higher temperatures may also mean that less frequent reheating is required, leading to reduced energy consumption. Environmentally, this can result in a decrease in greenhouse gas emissions, aligning with global efforts to combat climate change.

Lastly, for the professionals on the ground, a mix that remains workable at elevated temperatures might reduce the necessity for rapid, high-temperature operations, thereby promoting a safer and more manageable working environment.

In essence, the judicious introduction of SMR into AP-5 asphalt not only modifies its thermal profile but also offers a spectrum of potential benefits that are economically, environmentally, and ergonomically advantageous in practical scenarios.

### 3.6. Differential Scanning Calorimetry (DSC) Analysis of Shredded Mask Residues in Base AP-5 Asphalt

Utilizing the differential scanning calorimetry (DSC) method—a keystone in thermal analysis—we sought to elucidate the intricate ramifications of varying concentrations of shredded mask residues (SMRs) on the thermal behavior and phase transitions inherent in base AP-5 asphalt. Our investigative focus was honed on concentrations of 3, 6, and 9 wt. % SMR.

**Figure 9:**
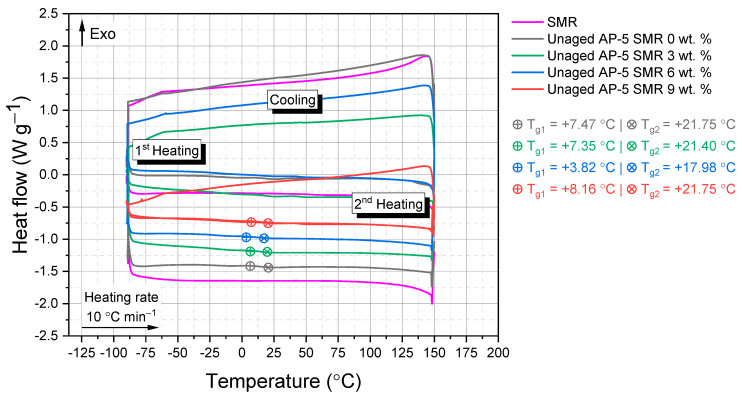
DSC thermograms showcasing the thermal profiles of fresh–virgin base AP-5 bitumen, shredded mask residues (SMRs), and AP-5 formulations integrated with varied proportions of SMR (e.g., 3, 6, and 9 wt. %).

From Figure 9, distinct thermograms of both unmodified AP-5 asphalt and its counterparts enriched with SMR emerge. In parallel, this figure presents a detailed heat flow thermogram exclusively for SMR. Comprehensive scrutiny of these thermograms uncovers that the fresh–virgin base AP-5 asphalt exhibits two pronounced glass transition temperatures: T_g1_ = +7.47 °C and T_g2_ = +21.75 °C. These dual T_g_ phenomena underscore the asphalt’s inherent heterogeneity, which is commonplace in many paving grade bitumina. Within the asphalt’s amorphous construct, the T_g_ elevation closely intertwines with the molecular aspects like polarity, aromaticity (i.e., surpassing a significant threshold, the aromatic constituency in base AP-5 asphalt embodies around 59.52 wt. % of its framework), structural rigidity, and the repetitiveness of molecular formations [85,86].

The T_g_ of neat AP-5 asphalt, much like other asphalts, may differ from values published in the broader academic sphere [87,88,89,90]. This variability in T_g_ is intrinsically tied to the asphalt’s geological origins, production methodologies, and compositional variances [87,88,89,90]. Geological influences, marked by specific diagenetic processes and the nature of primary organic matter, contribute to these unique T_g_ values. Refining nuances, from precise distillation techniques to custom fractionation and storage conditions, further nuance this T_g_ framework. Given this tapestry of determinants, the observed spectrum of T_g_ in conventional asphalts in the literature is both expected and corroborated [87,88,89,90].

These phase transitions are rooted in diverse amorphous configurations within the asphalt’s fabric. They are derived from: (1) Saturated compounds, chiefly linear alkanes or elongated aliphatic structures, marked by a T_g Sat_ between −88 °C and −60 °C. (2) The maltene domain, an amalgamation of saturates, aromatics, and resins, signaling a T_g Mal_ at approximately −20 °C. The maltene–asphaltene interface (3), possibly abundant in resins, shows a T_g Mal–As_ near −10 °C. Finally, (4) asphaltenes—characterized by alkylated and condensed aromatic structures—have a T_g As_ around +70 °C [91,92,93,94].

The SMR, synthesized from synthetic polymers across three distinct layers of face KF94 masks, introduces additional complexities in thermal transitions. The masks, rich in resins (100 wt. %), leverage their inherent thermal properties to possibly affect the asphalt’s T_g_. An in-depth analysis of Figure 9 indicates that as the SMR is incrementally blended into the asphalt, an additional glass transition emerges. This transition spans 3.82–8.16 °C for T_g1_ and 17.98–21.75 °C for T_g2_, likely stemming from interactions between the maltene phase of asphalt and the polymers in SMR, notably the resin components.

SMR’s unique polymeric makeup translates to distinctive thermal behavior. Figure 9’s DSC curve for SMR suggests no discernible glass transition temperatures within the examined range—a testament to the complex polymeric structure of the mask layers. Such intricacy offers challenges and opportunities when integrated with asphalt.

In application, these thermal dynamics dictate asphalt’s workability, durability, and longevity. A decrease in T_g_ might enhance its flexibility and resistance to cold-induced cracking [95,96], while variations in other thermal parameters could influence its resistance to deformations like rutting.

Ultimately, deciphering the thermal adaptations triggered by SMR in AP-5 asphalt not only unveils the microscopic interactions but also has far-reaching implications for infrastructure. As society grapples with waste management, redirecting discarded items like masks into infrastructural resources could pave the way for sustainable urbanization.

### 3.7. An Evaluation of Base AP-5 Asphalt’s Rheological Properties Influenced by Varied Concentrations of Shredded Mask Residues: An Analytical Approach Using Binder Empirical Tests

In this comprehensive research endeavor, we delved into the intricate effects of integrating varying proportions of shredded mask residues (SMRs) into base AP-5 asphalt. Utilizing fractions of 3, 6, and 9 wt. % SMR, this study meticulously scrutinized the resultant alterations in the physical attributes of the asphaltic binder. Conducted under unaged conditions, the analytical framework employed pivotal conventional binder tests, encompassing the penetration, softening point, viscosity, and ductility. The outcomes aim to elucidate the potential feasibility and efficacy of SMR as a modifying agent in the realm of asphalt material science.

#### 3.7.1. Penetration Analysis in the Presence of Shredded Mask Residues in Base AP-5 Asphalt

The intricate dynamics between asphalt’s rheological properties and its constituent compounds form the bedrock of our understanding of its performance in infrastructural applications. In our endeavor to ascertain the effects of shredded mask residues (SMRs) on the penetration value of fresh–plain base AP-5 asphalt binder, a comprehensive examination was undertaken.

**Figure 10:**
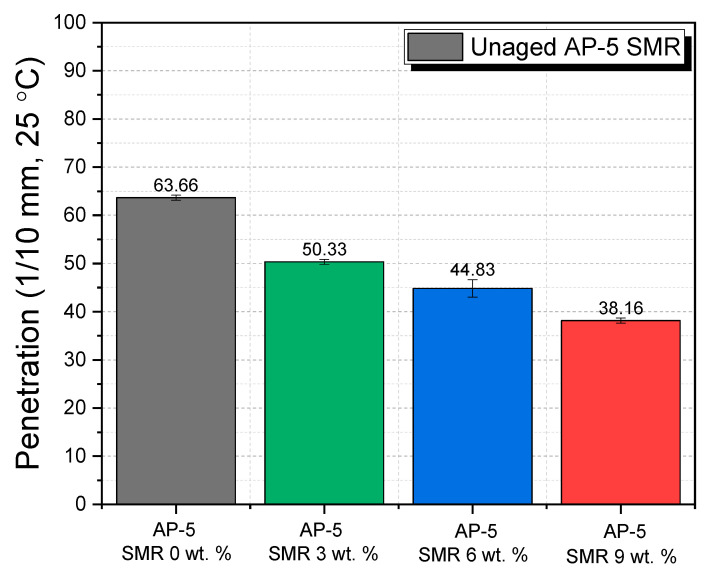
Impact of varied proportions of shredded mask residues (e.g., 3, 6, and 9 wt. % SMR) on the penetration traits of pristine base AP-5 asphalt binder.

It is understood that penetration properties play an imperative role in predicting the functional behavior of asphalt binders. Notably, these properties expound on the binder’s consistency and hardness, two quintessential parameters that delineate its aptness for road construction. With the incremental inclusion of SMR (at concentrations of 3, 6, and 9 wt. %), a noteworthy diminution in the penetration value of AP-5 asphalt was observed in Figure 10. As substantiated by the data, the pristine AP-5 asphalt exhibited a penetration value of 63.67 ± 0.51 dmm, while with the addition of 3, 6, and 9 wt. % SMR, the values registered were 50.33 ± 0.51, 44.83 ± 1.80, and 38.16 ± 0.51 dmm, respectively.

This trend evinces a direct correlation: with rising SMR concentrations, there is a concomitant decrease in the asphalt’s penetration value, a finding that is consonant with other research endeavors in this domain [23,46,97]. While one might attribute this to potential chemical interactions given that SMR is fundamentally considered a 100 wt. % resin, our Fourier-transform infrared spectroscopy (FT-IR) results present an intriguing revelation. The SMR does not instigate any significant chemical alterations within the binder. Instead, its influence is predominantly physical, akin to the role of a filler.

By acting as fillers, the SMRs significantly reduce the interstitial void spaces in the asphalt, leading to a denser matrix. Such densification invariably accentuates the hardness of the binder, culminating in reduced penetration values. From a molecular standpoint, the SMR, given its resinous nature, fortifies the internal structure, making the binder less pliable, yet potentially more resistant to forms of deformation such as rutting.

Translating this to real-world implications, a binder with lower penetration values, fortified with SMRs, might exhibit superior rutting resistance, especially in regions subjected to high vehicular stresses. However, a cautionary note is necessary here: while higher hardness could be advantageous in terms of rut resistance, it may compromise the binder’s flexibility and its ability to cope with thermal fluctuations, potentially leading to cracking.

In essence, the incorporation of SMR into the AP-5 asphalt has unveiled a potential avenue for producing denser, harder binders. Yet, it underscores the perpetual balance that must be struck between hardness and flexibility, ensuring longevity and functionality in road construction. Future endeavors should aim to delve deeper into the long-term field performance of such modified binders, weighing their benefits against the possible drawbacks in diverse climatic and traffic scenarios.

#### 3.7.2. The Enhanced Thermal Resistance of Base AP-5 Asphalt via Incremental Shredded Mask Residue Integration

The softening point of asphalt binders provides pivotal insights into their thermal behavior, particularly into the temperature at which the binder transitions from a relatively solid state to a viscoelastic state. This transition temperature, denoted as the softening point, is imperative in determining the binder’s resistance to high-temperature deformations, which is a crucial characteristic, especially for roadways exposed to intense solar radiation or high ambient temperatures.

**Figure 11:**
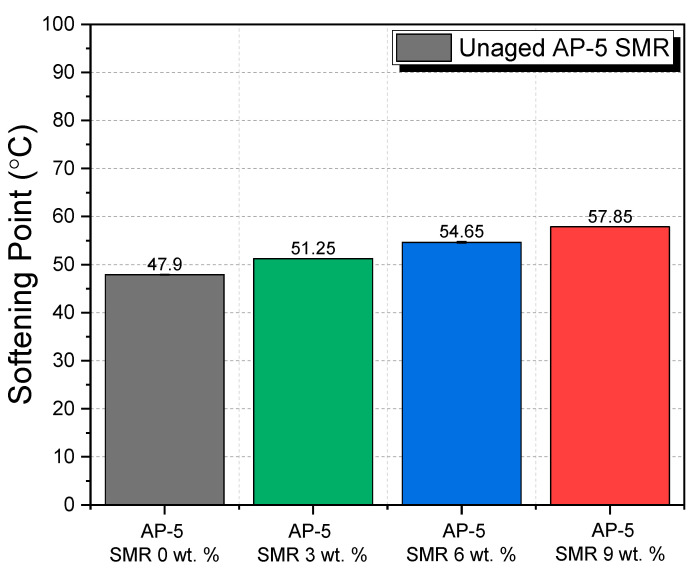
Modulation of softening point in unaged base AP-5 bitumen upon integration of varied proportions of shredded mask residues (e.g., 3, 6, and 9 wt. % SMR).

Our assessment pivoted on discerning the alterations in the softening point of the base AP-5 bitumen with incremental incorporations of shredded mask residues (SMRs) at proportions of 3, 6, and 9 wt. %. The data from Figure 11 indicated a discernible and consistent elevation in the softening point with increased fractions of SMR, a trend that aligns with outcomes previously chronicled in the literature [98,99,100]. Specifically, the softening point rose from 47.90 ± 0.11 °C for the pristine AP-5 binder to 57.85 ± 0.05 °C for the variant with 9 wt. % SMR. This increasing trend suggests an enhancement in the binder’s thermal stability and its resilience against deformations at high temperatures.

Delving into the chemical perspective, it is imperative to note that SMRs, being constituted of 100 wt. % resins (i.e., synthetic PP polymers), do not chemically interact or amalgamate with the bituminous matrix, as evidenced by the previous FT-IR analyses. Their role, rather intriguingly, is majorly physical, akin to that of a filler or stabilizer. The synthetic polymers from SMR, owing to their inherent thermal stability and rigidity, reinforce the binder matrix. Their presence seems to obstruct the easy flow of the asphaltene molecules, thereby elevating the temperature at which the binder begins to soften. This physical obstruction and reinforcement mechanism enhances the binder’s high-temperature performance, making it more resistant to rutting phenomena, which is a common distress in asphalt pavements.

In real-world scenarios, this elevation in the softening point suggests that roads constructed using AP-5 binders integrated with SMRs could offer superior performance in warmer climates. The heightened thermal stability ensures that the pavement retains its structural integrity even during peak summer months, reducing maintenance costs and ensuring vehicular safety. Moreover, the ability of the SMR-integrated binder to resist rutting can also extend the lifespan of the pavement.

In summary, the integration of shredded mask residues into the AP-5 asphalt binder not only provides an innovative means to repurpose waste but also significantly augments the thermal performance of the binder, presenting promising prospects for the construction of more resilient and sustainable roadways.

#### 3.7.3. Rotational Viscosity Modulation in Base AP-5 Asphalt with Incremental Shredded Mask Residue Integration

The determination of viscosity was meticulously pursued to understand the ramifications of incorporating diverse fractions (e.g., 3, 6, and 9 wt. %) of shredded mask residues (SMRs) on the viscosity metrics of the AP-5 asphalt binder. Viscosity, as a measure of a fluid’s resistance to shear or flow, gives pivotal insights into the binder’s flow characteristics and workability. These attributes are paramount in ensuring the optimal performance and durability of asphalt mixtures when used in roadway infrastructures.

**Figure 12:**
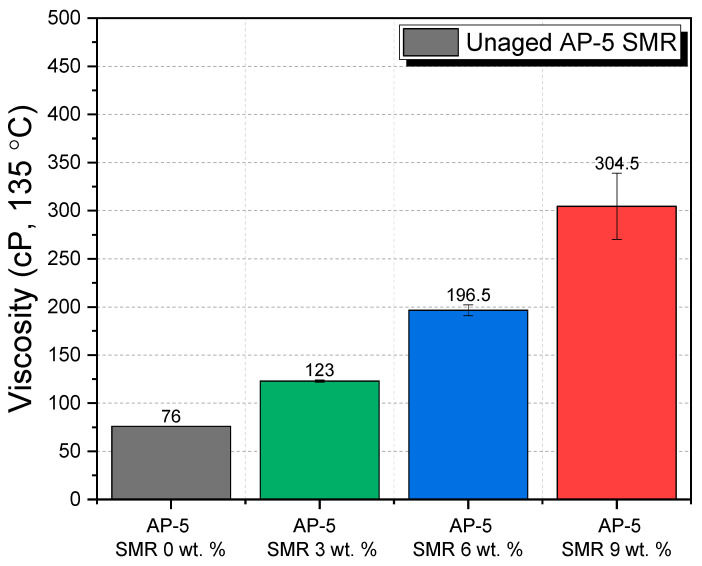
Effect of incremental shredded mask residues (e.g., 3, 6, and 9 wt. % SMR) on the rotational viscosity of unaged base AP-5 bitumen.

The analysis data from Figure 12 illustrates a prominent escalation in viscosity concurrent with the increasing concentrations of SMR, mirroring trends documented in extant literature [23,97,101]. Specifically, the viscosity increased from 76.00 ± 00 cP for the control sample to 304.50 ± 34.48 cP for the 9 wt. % SMR sample, implying a four-fold augmentation. This observation underscores that the shredded masks, consisting of a mixture of synthetic polymers (i.e., resins), augment the internal resistance to flow within the asphalt binder, thereby amplifying the viscosity. It denotes a potential reduction in the asphalt’s ease of application and fluidity, factors essential during the laying phase of road construction.

Chemically, previous FT-IR analyses denote that SMRs do not instigate significant chemical alterations within the binder. Instead, their contribution to the heightened viscosity is predominantly physical. The inherent irregular morphology and non-bituminous constitution of these shredded masks lead to an intricate network of interparticle interactions, which challenges the unhindered flow of the binder. Consequently, this physical impediment increases the rotational viscosity, which could influence the mixture’s compaction efficacy and the ease of blending during road construction operations.

In real-world applications, a binder with increased viscosity might necessitate alterations in mixing and laying protocols to ensure the asphalt mixture’s performance remains uncompromised. Furthermore, an escalated viscosity might imply an enhanced resistance to rutting and deformation under traffic loads, given that the mixture demonstrates reduced fluidity.

Conclusively, while the integration of SMRs offers an avenue for waste management and the potential enhancement of certain asphalt properties, it underscores the importance of recalibrating construction processes and expectations in light of these alterations in binder rheology.

#### 3.7.4. Impact of Shredded Mask Residues on the Ductility of AP-5 Bitumen: Implications for Asphalt Binder Elasticity and Pavement Durability 

In asphalt research and road construction, the ductility of bitumen acts as an imperative metric, depicting the binder’s capacity to elongate without severance. This characteristic is indispensable, particularly in estimating the material’s resilience against fracture and deformation due to vehicular stresses and climatic fluctuations. Our endeavor here is to discern the effects of the incorporation of varying proportions (e.g., 3, 6, and 9 wt. %) of shredded mask residues (SMRs) on the ductility of AP-5 bitumen.

**Figure 13:**
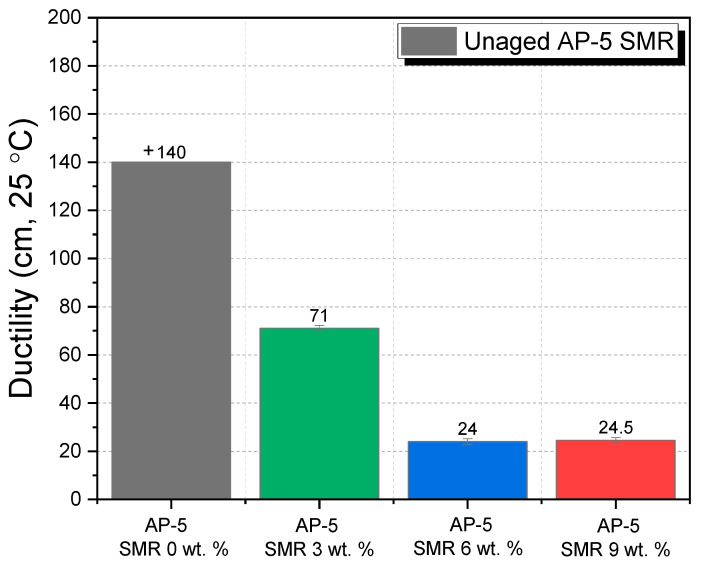
Variation in ductility of unaged base AP-5 bitumen influenced by specific fractions of shredded mask residues (e.g., 3, 6, and 9 wt. % SMR).

With an increase in the fraction of SMR, there is a distinct trend of decrement in the bitumen’s ductility—a trend robustly substantiated by ancillary literature findings [97,100]. The data from Figure 13 highlights that the ductility of AP-5 bitumen dips to almost half its original value with the inclusion of a merely 3 wt. % SMR, and further dips significantly at higher concentrations. These results underscore the fact that the integration of shredded masks impedes the asphalt binder’s elasticity, symbolizing an augmented susceptibility to fatigue and cracking.

From a chemistry perspective, the shredded masks—largely comprised of synthetic polymers or essentially 100 wt. % resins—do not seem to chemically alter the bitumen, as corroborated by FT-IR analyses. Yet, the physical implications are profound. The irregular morphology of the shredded masks and their non-bituminous compositions induce certain structural rigidity within the binder. This physically introduced restraint restricts the intrinsic stretching ability of the binder, causing a dip in its ductility. Essentially, SMR behaves akin to a filler, offering additional physical barriers within the bitumen, leading to decreased deformability.

In a real-world context, this decrement in ductility holds significant repercussions. Reduced ductility could be suggestive of a predisposition toward premature pavement distress, especially cracking under repeated traffic loads. Considering the critical role of ductility in ascertaining a pavement’s life span and overall performance, further research might be necessitated to strike a balance between the benefits and potential limitations of SMR integration in asphalt mixtures.

### 3.8. Catalyzing Asphalt’s Thermal Resilience: An Analytical Evaluation of Integrating Shredded Mask Residues (SMRs)

In the intricate realm of bituminous materials, the inherent thermoplastic properties of bitumen lead to a pronounced variability in consistency upon encountering thermal fluctuations. Such a phenomenon accentuates the criticality of the Temperature Susceptibility (TS) parameter when contemplating asphalt pavement design. This parameter fundamentally gauges the amplitude and direction of binder consistency alterations—whether expressed in terms of penetration or viscosity—under varying thermal conditions. For the purposes of this scientific investigation, we systematically deployed both the Penetration Index (PI) and the Penetration-Viscosity Number (PVN) as analytical tools, aiming to elucidate the TS characteristics of unaged asphalt specimens when amalgamated with graded concentrations of shredded mask residues (SMRs), specifically at 3, 6, and 9 wt. %.

#### 3.8.1. An Analysis of Penetration-Index (PI) in Unaged Base AP-5 Asphalt Integrated with Shredded Mask Residues

**Figure 14:**
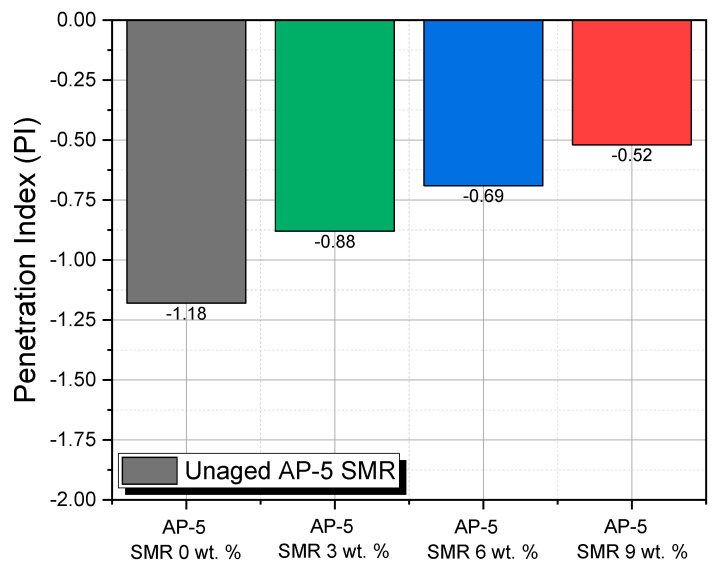
Influence of incremental concentrations of shredded mask residues (e.g., 3, 6, and 9 wt. % SMR) on the Penetration Index (PI) of unaged base AP-5 asphalt.

Upon scrutiny of the data encapsulated in Figure 14, one discerns that the PI values are stratified within a range demarcated by −1.18 and +0.5. From an infrastructural robustness perspective, it is axiomatic that, for an asphalted surface to proffer optimal functionality, the corresponding PI values ought to be nestled within the −1 to +1 interval [59]. When the PI values perilously approach or breach the −2 benchmark, one is confronted with an asphalt matrix that exhibits amplified thermal susceptibility [59]. Contextualized within the prevailing challenges posed by climate change, asphalts with such characteristics manifest an accentuated predilection towards undergoing brittle failures [59]. In a contrasting scenario, asphalts whose PI values transcend +1 often betray an inherent brittleness, a trait that is frequently concomitant with reduced thermal sensitivity coupled with enhanced elastic properties [59].

A notable observation from the data portrayed in the Figure 14 set pertains to the pristine AP-5 asphalt, devoid of any SMR integration, which registered the most diminished PI value at −1.18. Yet, a compelling trend emerges upon the incremental integration of SMR: a consistent and discernible ascent in PI values. Considering that SMR—sourced from multifarious layers of KF94 masks—boasts a predominant resinous constitution, amounting to 100 wt. % resins, its modus operandi within the asphalt matrix leans toward a filler capacity rather than assuming the role of a reactive additive. The observed PI trajectory, ascending with increasing SMR concentrations, firmly underscores the instrumental role SMR plays in attenuating the thermal susceptibility of the asphalt matrix.

From a pragmatic vantage point, this seminal research resonates with broader implications, especially when one contemplates the exigencies of modern infrastructural challenges, amplified by pronounced thermal gradients. The judicious integration of SMR not only heralds an avant-garde strategy for sustainable waste management in the aftermath of the global pandemic but also portends the optimization of asphalt’s functional properties. Thus, this research serves as a clarion call for intensified academic pursuits at the intersection of sustainable waste valorization and infrastructural resilience.

#### 3.8.2. An Analysis of the Penetration-Viscosity Number (PVN) of Unaged Base AP-5 Asphalt Integrated with Shredded Mask Residues

**Figure 15:**
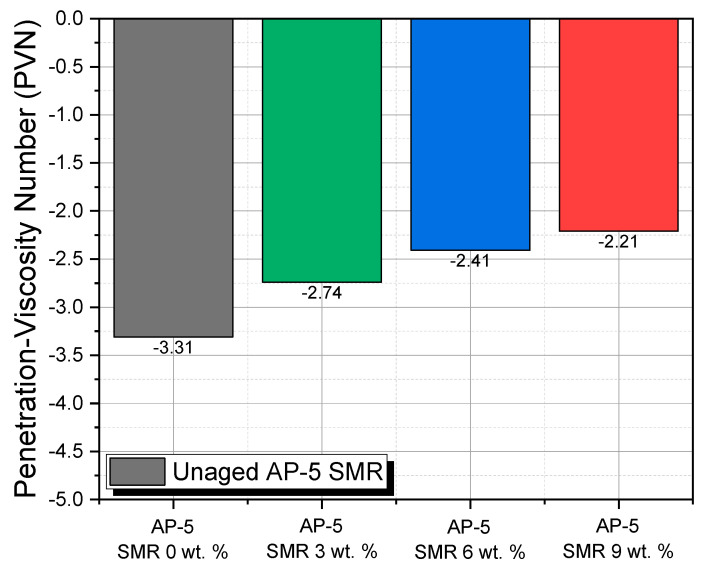
Influence of graded concentrations of shredded mask residues (e.g., 3, 6, and 9 wt. % SMR) on the Penetration-Viscosity Number (PVN) of virgin base AP-5 asphalt in its unaged state.

Figure 15 delineates the computed PVN values, highlighting a range from −3.31 to −2.21 for unaged AP-5 asphalt samples at various fractions of SMR integration—namely 3, 6, and 9 wt. %. It is pertinent to note that paving grade asphalts conventionally have PVN values oscillating between −2.0 and +0.5. Higher PVN values signal a reduced temperature sensitivity of the asphalt binder [59].

Within the context of the present research, the pristine AP-5 asphalt exhibited a PVN of –3.31, outlining a substantial margin for improving its thermal susceptibility. It was observed that with a progressive increase in SMR concentration, there was a notable elevation in the PVN values, indicating an enhancement in the thermal susceptibility. The SMR, essentially comprising 100 wt. % resins derived from the comprehensive layers of KF94 masks, functioned as a non-reactive filler, rendering stability against temperature-induced variations.

Examining the real-world implications, this augmentation in PVN values underscores a promising pathway in enhancing the resistance of road pavements to adverse weather conditions, specifically cold environments prone to causing material brittleness and subsequent transverse cracking. The highest PVN value was recorded at a 9 wt. % SMR integration, establishing a benchmark that the additive can indeed foster better resilience in the AP-5 asphalt matrix even under unaged conditions. It should be underlined that the introduction of SMR not only represents a strategy to bolster the performance characteristics of asphalt but also embarks upon a sustainable route, leveraging waste materials effectively.

It is therefore inferred that the strategic inclusion of SMR could significantly better the thermal susceptibility profile of the AP-5 asphalt, demonstrating its potential for deployment in regions experiencing colder climates. Future studies could potentially explore a varied range of SMR concentrations to fine-tune the optimal balance between thermal susceptibility and other desirable asphalt properties.

In conclusion, the introduction of shredded mask residues in fresh AP-5 asphalt formulations shows a remarkable prospect for optimizing pavement materials while championing a sustainable approach to waste management, opening avenues for robust and environmentally conscious infrastructural developments. This study paves the way for further investigations into leveraging shredded mask residues to enhance asphalt properties, heralding a new epoch in road construction technologies grounded in sustainability and innovation.

### 3.9. The Dynamic Shear Rheometer (DSR) Analysis of Base AP-5 Asphalt Modified with Shredded Mask Residues: Implications for Rutting Behavior

In asphalt rheology, the Dynamic Shear Rheometer (DSR) stands as an imperative tool, allowing for nuanced understandings of binder behaviors under shear stresses. This investigation utilized the DSR to elucidate the ramifications of incorporating varying fractions of shredded mask residues (SMR at 3, 6, and 9 wt. %) on the rutting propensity of virgin base AP-5 asphalt. The evaluation metric, the rutting resistance factor, expressed as (G*/sin δ), where |G*| demarcates the stiffness or complex shear modulus, and “δ” is the phase angle, was pivotal in gauging shear deformation tendencies. An elastic binder with heightened stiffness—reflected through augmented (G*/sin δ) values—stands as the paragon for superior rutting resistance. For unaged asphalts post-construction, it is imperative that the stiffness measure of G*/sin δ surpasses 1.0 kPa at the operational temperature, ensuring their robustness against tenderness during the mixing, placement, and compaction phases [102].

**Figure 16:**
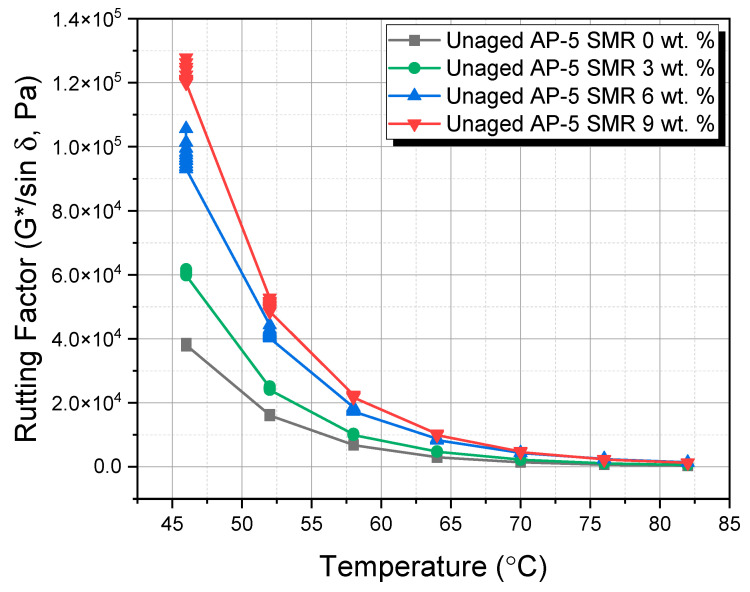
Comparative analysis of rutting factor (G*/sin δ) as a function of temperature in unaged base AP-5 asphalt and formulations containing diverse concentrations of shredded mask residues (e.g., 3, 6, and 9 wt. % SMR).

From Figure 16, the baseline AP-5 asphalt (labeled as Unaged AP-5 SMR 0 wt. %) was found to exhibit minimal rutting susceptibility when juxtaposed with SMR-modified specimens. Intriguingly, with the incremental addition of SMR, the stiffness parameter (G*/sin δ) demonstrated a consistent uptrend across diverse temperature spectra. Such a phenomenon is attributable to the innate characteristics of SMR—chiefly its polymers derived from various KF94 mask layers. These polymers, acting as non-reactive fillers, imbue the asphalt matrix with enhanced cohesion, inherently bolstering its rutting resistance.

A meticulous juxtaposition with traditional testing techniques revealed that increasing the SMR concentrations led to a notable decrease in the penetration and ductility of the asphalt binder. Simultaneously, a discernible increase in both the softening point and the viscosity was observed. The heightened softening point suggests an augmented thermal threshold for the binder, implying a reduced risk of rutting at elevated temperatures. Moreover, the increased viscosity typically intimates a more viscous, less fluid binder, beneficial in resisting deformation under traffic loads. Conversely, the diminished penetration and ductility indicate a reduced ability of the binder to flow and elongate—properties crucial for low-temperature performance.

In a temperature regime below 70 °C, all asphalts—irrespective of their SMR composition—complied with the stringency of Superior Performing Asphalt Paving (Superpave) Specifications, registering a stiffness of G*/sin δ ≥ 1 kPa [102]. Explicitly, at temperature markers of 70, 76, 82, and 82 °C, both the unmodified AP-5 asphalt and its SMR-infused variants (i.e., 3, 6, 9 wt. %) met the stipulated standards.

To encapsulate, the research delineates a transformative avenue in asphalt technology. The conscientious utilization of pandemic-induced waste, typified by discarded masks, amalgamates environmental prudence with infrastructural excellence. By judiciously integrating SMR into asphalt, there emerges a duality of benefits: environmental sustainability dovetailed with enhanced road surface performance, particularly concerning rutting resistance.

## 4. Conclusions

In the rapidly evolving landscape of global infrastructure, particularly amidst the backdrop of pandemics, sustainable and innovative solutions are paramount. The present study, marked by its novelty, delves deep into the utilization of mask waste—an inevitable byproduct of our time—as an asphalt modifier with potential ecological benefits and improved pavement durability.

Through an exhaustive experimental regime, fresh–virgin base AP-5 asphalt was modified with varying fractions of sterilized shredded mask residues (SMRs) ranging from 3 to 9 wt. %. The applied methodologies were robust, encompassing a broad spectrum of tests from chemical, to physical, to rheological analyses using cutting-edge techniques. Notably, the study leveraged TLC-FID, FT-IR, SEM, TGA, and DSC methods, among other techniques, ensuring a comprehensive understanding of the resultant asphalt mixtures.

The chemical analyses, specifically via the TLC-FID, divulged a nuanced understanding of the composition post-SMR addition. While saturates remained fairly stable, a slight decrement in aromatics was observed. Worth noting was the significant increase in resins, which, coupled with a decrease in asphaltenes, underscored the rejuvenating impact of SMR on the asphalt. This effect was further corroborated by the enhanced thermodynamic stability as indicated by the colloidal stability index (I_C_).

From a structural standpoint, the FT-IR findings illuminated that SMR predominantly acted as a filler, rather than instigating any extensive chemical reaction. This insight is paramount, as it establishes the role of SMR in enhancing binder properties without majorly altering the chemical composition of the asphalt.

The thermal analyses were particularly revealing. The TGA evidenced a heightened initial temperature of degradation, affirming the thermal resilience of the modified asphalt. The DSC echoed these sentiments, elucidating an enhanced thermal performance, especially at lower temperatures.

The morphological insights gleaned from the SEM were critical. Higher dosages of SMR induced a rougher microsurface. However, an optimal dosage of a 3 wt. % SMR emerged as the frontrunner, evidenced by a harmonious amalgamation of lesser microtexture roughness and optimal assimilation with asphalt.

The rheological evaluations further enriched our understanding. A gradual decrease in the penetration and ductility was offset by gains in the softening point and viscosity, signaling an enhanced resistance to deformation and a superior binder performance. The findings from the dynamic shear rheometer (DSR) were especially salient, fortifying the premise that SMR can be a formidable adversary against rutting distress—one of the primary distresses plaguing road infrastructures.

Drawing from these results, an optimal dose of a 3 wt. % SMR for asphalt modification emerges as a recommendation, balancing the performance enhancements with the optimal material assimilation.

Future endeavors could explore the long-term aging characteristics of SMR-modified asphalts and their resilience against external environmental stressors. Additionally, field trials would be invaluable to ascertain real-world performance, especially under varied climatic conditions.

In summary, this investigation carves out a pioneering path in the realm of sustainable infrastructure solutions, offering a pragmatic approach to not only enhance road pavement durability but also address the looming environmental concerns of mask waste.

## Figures and Tables

**Table 1:** KF94 Mask: Components, material composition, and functional overview [41,42,43].

Components	Composition	Function
Outer layer	Non-woven polypropylene fabric	First barrier against larger external particles and droplets.
Filter layers	Polypropylene filter non-woven fabric	Filters out fine particles, including dust, pollutants, and microorganisms.
Inner layer	Soft non-woven polypropylene fabric	Provides comfort, absorbs moisture from breathing and sweating.
Nose bridge	Plastic with malleable metal strip	Ensures snug fit around the nose, minimizing unfiltered air entry/exit.
Straps	Polyurethane–Nylon	Secures the mask to the face, ensuring a tight seal.
Shape and design	Unique 3D design	Ensures snug fit and covers nose, mouth, and chin for effective protection.

**Table 2:** Detailed physicochemical profile of shredded mask residues (SMRs) derived from used KF94 masks.

Elemental Analysis	Mean ± SD
C (Carbon)	85.97 ± 0.31 wt. %
H (Hydrogen)	14.86 ± 0.05 wt. %
N (Nitrogen)	0.09 ± 0.00 wt. %
S (Sulfur)	0.00 ± 0.00 wt. %
O (Oxygen)	0.73 ± 0.04 wt. %
**SARA Generic Fractions**	
Saturates	0.00 ± 0.00 wt. %
Aromatics	0.00 ± 0.00 wt. %
Resins	100.00 ± 0.00 wt. %
Asphaltenes-like components	0.00 ± 0.00 wt. %
**Face Mask and Respirator Specifications** [43,49,50]	
Grade	KF94
Material	Polypropylene (PP, (C_3_H_6_)_n_)
Size	9.68 × 5.51 inches
Color	White
Filtration efficiency	≥94%
Total inward leakage rate	≤8.00%
Inhalation resistance	≤70 Pa (30 L min^−1^)
Exhalation resistance	≤120 Pa (85 L min^−1^)
Style	Protection–Safety
Reusability	Disposable
Country of origin	South Korea

**Table 3:** Comprehensive analysis of the physicochemical characteristics of base AP-5 asphalt cement.

Elemental Analysis	Mean ± SD
C (Carbon)	86.62 ± 1.62 wt. %
H (Hydrogen)	10.78 ± 0.19 wt. %
N (Nitrogen)	0.51 ± 0.02 wt. %
S (Sulfur)	5.65 ± 0.05 wt. %
O (Oxygen)	0.90 ± 0.03 wt. %
**SARA Generic Fractions**	
Saturates	3.47 ± 0.46 wt. %
Aromatics	59.52 ± 1.36 wt. %
Resins	16.30 ± 1.76 wt. %
Asphaltenes	20.67 ± 3.35 wt. %
Colloidal Instability Index (I_C_) ^†^	0.3184
**Physical Properties**	
Penetration at 25 °C	63.66 ± 0.51 dmm
Softening point	47.90 ± 0.11 °C
Rotational viscosity at 135 °C	76.00 ± 0.00 cP
Ductility at 25 °C	≥140.00 ± 0.00 cm
Density at 25 °C	1.00 ± 0.00 g cm^−3^

^†^ I_C_ = ([Saturates] + [Asphaltenes])/([Aromatics] + [Resins]).

**Table 4:** Thermogravimetric analysis metrics: A comparative assessment of shredded mask residues (SMRs), unmodified base AP-5 asphalt, and formulated AP-5–SMR blends with 3, 6, and 9 wt. % SMR, subjected to a consistent heating rate of 20 °C min^−1^.

Sample	TGA/DTGA (°C)						−ΔW (wt. %)
	Phase 1	Phase 2	Phase 3	T_onset_	T_offset_	T_max_	
SMR	36.02–364.95	364.95–427.36	427.36–999.96	364.95	427.36	409.95	1.49
AP-5 SMR 0 wt. %	36.15–378.55	378.55–463.62	463.62–999.95	378.55	463.62	441.21	0.63
AP-5 SMR 3 wt. %	35.48–439.04	439.04–461.81	461.81–999.95	439.04	461.81	386.37	0.48
AP-5 SMR 6 wt. %	35.95–402.51	402.51–468.51	468.51–999.95	402.51	468.51	448.33	2.52
AP-5 SMR 9 wt. %	35.88–415.74	415.74–475.72	475.72–999.95	415.74	475.72	454.16	10.83

T_onset_: Initial temperature indicative of thermal degradation (°C); T_offset_: Temperature marking the completion of thermal loss (°C); T_max_: Peak temperature of decomposition (°C); ΔW: Carbonaceous residue mass fraction at 1000 °C (wt. %).

## Data Availability

Data from this study can be accessed upon request by reaching out to the first author.
